# Beyond the Finish Line: Examining the Role of Children in Marathon Races—A Narrative Review

**DOI:** 10.3390/jfmk9010047

**Published:** 2024-03-07

**Authors:** Gerasimos V. Grivas

**Affiliations:** Physical Education and Sports, Division of Humanities and Political Sciences, Hellenic Naval Academy, 18539 Piraeus, Greece; grivasger@hotmail.com

**Keywords:** children, marathon, health risks, musculoskeletal conditions, cardiovascular system, training, psychological effects

## Abstract

Compared with other sports, running is popular sport for children throughout the world. Over the last few decades, marathon running has become increasingly popular even in the age group below 18 years. While the majority of youth athletes fall within the 16–18 age range, it is noteworthy that there are also participants younger than 12 years engaging in marathon races. Advice on the safety of youth athletes participating in these events is frequently sought by parents, coaches, sport scientists, and medical professionals, particularly concerning potential short- and long-term health consequences. The act of marathon running has the potential to impact key organ systems during the crucial phases of growth and development. To ensure the safety of marathon running in youth runners, it is essential to address multiple physiological and psychological aspects of health. These recommendations are directed towards ensuring the safe participation of youth athletes in marathon races through proper and individualized assessments.

## 1. Introduction

Physical inactivity is a risk factor for cardiovascular and other chronic diseases, including obesity, diabetes, hypertension, and cancer [1]. Recent health promotion guidelines for children recommend accumulating a minimum of 60 min of physical activity daily as part of transportation, physical education, sport, free play, and planned exercise [2]. Compared with other sports, running is popular sport for children throughout the world [3]. In 2012, running ranked as the second most common physical activity among boys aged 12–15 years (33.5%) and the most common among girls in the same age group (34.9%) in the United States [4]. Many studies are showing the beneficial effects of running on aerobic capacity, lipid profile, body composition, and blood pressure [5].

Over the last few decades, marathon running has seen a surge in popularity, even among individuals under 18 years of age [6]. Therefore, it is not surprising that thousands of youth marathoners participate in the Los Angeles Marathon. From 1982 to 2007, nearly 300 youth runners completed the Twin Cities Marathon, achieving finishing times ranging from 2 h:53 min to 6 h:10 min [7]. The youngest participant in these datasets was 7 years old [7]. Only four documented medical encounters from this race involved children (all minor in nature involving two runners aged 15 years and two runners aged 16 years). A similar example is that recently, on May 2022, a 6-year-old boy made national headlines after finishing the Flying Pig Marathon in Cincinnati, Ohio, in 8 h:35 min [5].

In the 1970s, numerous children achieved remarkable feats by completing marathons, with age-group records established for participants as young as 5 years old. Astonishingly, children as young as 8 completed the marathon race in an impressive 3 h:31 s [8]. Many adult marathoners have trained at high levels for years without reaching this mark. Despite the absence of reported injuries or adverse events, concerns regarding potential dangers of youth participation surfaced, prompting physicians and marathon race directors to voice apprehensions. This growing unease led to significant changes in marathon regulations. By 1981, a minimum age requirement of 16 years was enforced for participation in the New York City Marathon, with similar guidelines adopted in other locations [8]. It is noteworthy that these age restrictions were primarily implemented for administrative purposes and lacked a foundation in outcomes data. In 2005, approximately 1769 youth started the marathon race and 1744 finished (including 78 children aged 11 years), with no medical encounters reported [7].

On the other hand, Rice and Waniewski [9] expressed their concerns in an article entitled “Children and marathoning: how young is too young?”, and reported that children are not small versions of adults. Their anatomy and physiology are still developing and are not yet fully mature. Despite these well-established concepts, which are intuitively understood in a general sense, they are often forgotten or ignored in practice, particularly in athletic pursuits. Thus, from the above, the most commonly asked questions are the following: Can children run marathons?; What are the health consequences? The purpose of this review is to examine the existing knowledge regarding the potential impact of youth marathon runners preparing for and participating in marathon races on different organ systems based on the available evidence.

In this review, terms such as youth, children, boys, and girls represent different age ranges. According to the WHO [10], a youth group is an age-specific group that typically engages young people aged 15–24 years old. Children refer to both boys and girls under the age of 18 years old, while boys and girls specifically denote males and females under the age of 18 years old, respectively [10].

## 2. Methods

For this review, I searched the PubMed, SPORTDiscus, and MEDLINE databases to identify English-language sources using the following terms: “children”, “distance runners training”, “health risks”, “musculoskeletal conditions”, “psychological effects”, “training programs”, “cardiovascular system”, “recommendations for coaches”, AND “youth marathon runners”. I also searched the bibliographies of the retrieved articles. I included only English-language articles, with no publication time limit.

## 3. Health Risks

### 3.1. Musculoskeletal Conditions

Participation in any physical activity, especially marathons, elevates the risk of acute or overuse musculoskeletal injuries and, albeit rarely, medical collapse [5,11,12,13]. Numerous risk factors unique to growing children exist. Stress fractures, a distinct overuse injury, are well documented to be a function of both the number of repetitions and the applied force per repetition [11]. A child with a shorter stride length may experience a greater number of impact repetitions to cover the same distance as an adult. Immature articular cartilage, being more susceptible to shear force compared to adult cartilage, can predispose children to conditions such as osteochondritis dissecans [14]. For youth runners, the stage of growth and development may be a more appropriate measure of risk for injury or illness than chronological age [5].

Several published reports have highlighted stress-related injuries in youth athletes [7]. A comprehensive literature review identified 32 cases of physeal injury among youth athletes, with only two case studies involving long-distance runners—one pertaining to proximal tibial and the other to metatarsal metaphyseal widening [15]. Most of the school runners reported a history of running-related injuries. The most common injuries were plantar fasciitis, iliotibial band syndrome, and Osgood–Schlatter disease [16]. Additionally, a study by Goldman et al. [17] found that during a 28-week marathon training program, 18% of adolescent participants reported an injury. The most commonly reported injury site was the knee (33%), followed by the lower leg (19%), foot (14%), ankle (13%), thigh (6%), and hip (6%). Previous studies reported that runners with a history of running-related injuries tended to run greater average weekly mileage and ran faster [16].

### 3.2. Psychological Effects

One of the principal areas of concern in evaluating the effects of marathon training on youth athletes is the psychological effects [18]. Attention has predominantly centered on burnout, with children who engage in rigorous training during their youth often dropping out of the sport in their late teens [18]. This phenomenon is seen when looking at youth runners. The vast majority of successful top-ranked youth runners do not continue on to become top-level senior runners [18]. More especially, athletes immersed in intensive athletic endeavors often undergo emotional burnout and a decrease in self-esteem, leading to a waning interest in the very activity that dominated their childhood and early adolescent years [9]. The American Academy of Pediatrics’ Committee on Sports Medicine has identified psychological pressures as potential issues associated with distance running [19]. Intensive training at a young age may subject some children to psychological factors and burnout, ultimately leading to dropout from sport participation [7]. In Roberts’ review [7], it was reported that the majority of child athletes in all sports drop out by the age of 13 years. Despite the common trend of youth and high school athletes retiring after high school, the post-high school status of participants in distance running remains unknown. Also, the International Amateur Athletic Federation of England guidelines state that intense training in children can cause unnecessary phycological stress [20].

### 3.3. Cardiovascular System

It is well known that regular activity can reduce the risk of cardiovascular disease [21,22]. The ongoing debate revolves around whether prolonged endurance exercise contributes to chronic cardiovascular disease and increased mortality risk [22]. To our knowledge, no data are available on the impact of marathon on the cardiovascular system in young adults.

As youth athletes undergo growth and engage in endurance training, cardiac remodeling takes place, encompassing physiological, structural, and functional myocardial adaptations in all four chambers of the heart [23,24]. While there are no specific data on the effects of marathon training or racing on cardiac changes in youth athletes, notable heart remodeling has been observed in boys aged 14–18 training for 10 h per week in triathlon, cycling, or rowing [25,26]. The observed cardiac changes included left ventricular dilatation and/or physiological myocardial hypertrophy in 18% and 5% of athletes, respectively [25,26]. While incidents of sudden cardiac death during exercise are documented in youth athletes, no recorded instances of cardiac-related fatalities have been observed in children participating in marathon races. Evaluating the long-term implications of repeated minor cardiac stress from marathon race participation is crucial [27].

A few studies have examined the cardiovascular system of youth athletes in cycling and cross-country skiing. Five athletes under the age of 18 and 10 adult finishers participated in a 21-day ultra-endurance cycling ride covering a total distance of 3515 km, with an average of 167 ± 72 km per day. The study, conducted during the event, revealed no cardiac problems in the youth riders, while the adults showed an increase in significant arrhythmias in the 24 h Holter monitoring [28]. Another extensive cohort study, involving 6258 cross-country skiers aged 15–24, similarly found no observed cardiac problems [29].

On the other hand, numerous studies have investigated the effects of marathon races on the cardiovascular system in adult marathon runners. Over the past 40 years, the number of participants running in a marathon race has risen 20-fold. In 2010, an estimated half-million runners completed a marathon in the United States [30]. Sudden cardiac death among marathon runners is very rare, with 1 event per 100,000 participants [30,31]. While the per-participant risk has remained constant over the decades, the absolute mortality rates have increased alongside the growing number of participants [30]. The final 1.5 km of the marathon race comprises less than 5% of the total distance of 42.195 m, yet it is responsible for nearly 50% of the sudden cardiac deaths that occur during the race [32].

The most common causes of sudden cardiac death during or after extreme exertion in adults younger than 30 years typically involve genetic factors, such as hypertrophic cardiomyopathy, anomalous coronary arteries, dilated cardiomyopathy, and congenital long syndrome. In athletes aged 30 years and older, coronary heart disease, acute myocardial infarction, and ischemia are primary contributors to exercise-related sudden cardiac death [33,34].

### 3.4. Training in Youth Runners (Ages 12–18)

Marathon races are characterized by an intensity below the anaerobic threshold and entail extensive training volumes [35,36]. Youth runners typically engage high training volumes, averaging around 57 km per week [37], exceeding the reported weekly volume of 22–27 km for high school cross-country runners [38]. While Krabak et al. [39] reported limited evidence suggesting that the running volume observed in high school cross-country runners may pose a risk factor for running-related injuries, the association between running volume and injuries in youth marathon runners remains unknown [40].

Around the age of 7 or 8, children who have an interest in running may begin participating in fun runs and organized track and field programs that typically last a few months each year. It is beneficial for aspiring distance runners to engage not only in middle-distance races but also in sprinting, jumping, and throwing events during this time [41]. Following the track season, it is important for them to diversify their activities by taking part in sports like soccer, basketball, and other youth sports that they enjoy. Encouraging participation in multiple sports is crucial for developing overall physical fitness before starting specialized training for track and cross-country events [41].

Youth runners under the age of 12 years are notably considered a high-risk group due to their immature musculoskeletal system [42] and the critical age of psychosocial development [43]. By the ages of 12 and 15 for most girls and boys, respectively, key developmental changes have typically occurred, enabling them to safely begin a low-mileage, low-intensity training regimen. This approach allows for gradual improvement over time. It is important to note that this does not imply that younger children should completely avoid participating in distance running [41]. Instead, the suggestion is to defer specialized year-round training. Furthermore, runners in this age group (12–15 years old) are classified as medium risk due to their psychological maturity, capacity to provide informed consent, and comparatively more developed musculoskeletal systems [44]. Youth runners aged 16–18 years are classified as low risk due to the maturation of their organ systems. This age category has demonstrated the highest success rates in completing marathon races [33].

The sports medicine community has responded to the growth in youth running participation by generating two consensus statements. The first international consensus statement, published in 2021 [39], was followed by a later consensus statement specifically addressing the safety of youth runners completing ultra-endurance events, typically spanning 50–161 km [40]. Both expert groups acknowledged the low level of evidence for reaching definitive conclusions across most health domains. Despite this, both documents underscore the recognized benefits of youth sports and offer practical recommendations. These guidelines aim to assist coaches and researchers in conducting individualized assessments to determine the appropriateness of participation in running events. According to Brenner et al. [45], in a review on pediatric overuse injury, there is no reason to prohibit participation in a well-organized marathon race, provided that the athlete derives enjoyment from the activity and remains asymptomatic.

### 3.5. Strength Training

Core, hip, and lower-extremity muscle weakness may contribute to injury risk in the youth runners, although there is conflicting evidence in the literature. For instance, in a cohort of high school cross-country runners, weak hip abductors were associated with anterior knee patellofemoral pain syndrome [46]. However, a study of high school cross-country runners showed improved race times following a 6-week pelvic and core strengthening program [46]. Finally, a recent meta-analysis of runners showed discrepancy between cross-sectional and prospective studies of hip weakness and patellofemoral pain, suggesting that hip weakness may be secondary to patellofemoral pain in some cases [47].

Strength training offers numerous benefits for youth runners. These include an improved cardiovascular risk profile, better weight control, decreased sports-related injuries, improved psychological wellbeing, and improved motor performance skills [44,48]. Targeted spine and hip strengthening exercises may be effective for rehabilitation and/or reduction in common running acute and overuse injuries. These injuries include Achilles tendinosis, ankle sprain, hamstring strain, patellofemoral pain, iliotibial band syndrome, and tibial bone stress injury [49]. The above exercises should incorporate running-specific functional movements. They should progress from double-leg to single-leg squats and hops, and eventually to more demanding plyometrics [50].

### 3.6. Sex Differences

Based on sex differences, there is a lack of published studies comparing health-related outcomes in boys and girls who complete marathon races. For instance, a 15-year prospective study of 3233 high school cross-country runners revealed higher injury rates among girls, with 16.7 injuries per 1000 athlete exposures, compared to 10.9 injuries per 1000 athlete exposures in boys [51]. Similarly, an 8-year prospective study of high school athletes found a higher rate of medical disqualification due to injury among girls compared to boys, with rates of 5.6 per 100,000 athlete exposures and 2.1 per 100,000 athlete exposures, respectively [52]. Moreover, a 20-year longitudinal study of middle school cross-country runners demonstrated higher injury rates and increased incidence of bone stress injuries [53,54]. Scheer et al. [40] reported that adolescent girls in cross-country face a greater risk of running-related injuries compared to boys. Longitudinal data also indicate a high lifetime prevalence of stress fractures among adults who participated in ultra-endurance running during their youth, although further research is needed [37].

To reduce the increased risk of injury among youth distance runners, it is recommended that they incorporate strength training, stretching, and foam rolling into their routine [55].

### 3.7. Footwear and Technique

There is a limited amount of research on running injuries in children concerning mechanics and footwear. On the other hand, studies in adults have shown that habitual running in minimal footwear with no cushioning or barefoot encourages a forefoot strike running pattern due to discomfort when landing on the heel [43,56]. This pattern offers various advantages, such as improved strength and stiffness of the Achilles tendon [57], reduced stress on the anterior shin [58], and decreased contract stress in the patellofemoral joint [59]. Additionally, Krabak et al. [50] reported that a forefoot strike pattern typically leads to an increased step rate, which, when raised by 7.5% to 10%, can significantly decrease load rates on the hip and knee running [60], while also correlating with a reduction in shin injuries among youth runners [61].

Many studies suggest that reduced shoe support may promote an increase in intrinsic foot muscle strength [62,63]. In the study by Rao and Joseph [64], the subjects are children and have shown that individuals who habitually go barefoot have a significantly lower incidence of pes planus compared to those who wear shoes. Moreover, adults who run in minimal shoes without support experience increased muscle size and presumed strength in the intrinsic and extrinsic foot muscles [65]. Conversely, runners using carbon fiber plate footwear have been observed to develop bone stress injuries [66]. Enhanced foot strength resulting from increased muscle size may offer protection against future foot and ankle injuries [65]. Overall, adopting a forefoot strike and increasing step rate have been associated with improved strength and decreased risk of common injuries in both youth and adult runners [58,63,67,68]. Perhaps starting youth runners in footwear that encourages a forefoot strike pattern may promote greater foot and ankle strength, potentially reducing the risk of future injuries [50].

### 3.8. Recommendations for Fitness Professionals or Practitioners and Coaches

The assessment and support of the training status and preparation for marathon races in youth athletes are crucial responsibilities of their coaches (Table 1). It is widely accepted and recommended that youth athletes across all age groups undergo examination before participating in competitive sports [40]. Notably, youth runners under the age of 12 years constitute a high-risk group, given their immature musculoskeletal system [42] and the critical age in their psychosocial development [40]. Youth athletes aged between 12 and 15 years are classified as medium risk due to their psychological maturity, ability to provide informed consent, and more developed musculoskeletal systems, though it is noteworthy that peak height velocity typically occurs during this age range [44]. Despite this, bone growth may still outpace the maturation of the muscle–tendon–bone complex in youth runners [45,69], posing a potential risk to the musculoskeletal system [50]. Therefore, careful assessment is essential when considering these athletes for marathon participation. The 16–18-year-old youth athlete group, in the transition to adulthood, is categorized as low risk due to the physical maturation of organ systems. This age category has demonstrated the highest success rates in completing marathon races [40].

Among adult marathon runners, the most common musculoskeletal injuries include anterior knee pain, iliotibial band friction syndrome, tibial stress syndrome, plantar fasciitis, Achilles tendonitis, and meniscal injuries of the knee [70].

Based on the available evidence, this review suggests a multifaceted approach for children aspiring to participate in marathon races (Table 1). Emphasis is placed on intrinsic motivation over external pressures, and children are empowered with the knowledge that they can cease participation at any point without facing repercussions. Parents are advised that marathon participation in childhood does not necessarily confer long-term competitive advantages. Instead, starting with shorter races and gradually progressing to longer distance is recommended [4], with readiness assessed based on individual growth and development [44,71]. Adequate rest, periodic breaks, and limited yearly participation are stressed to prevent overtraining and associated injuries. Nutritional supplementation with calcium and vitamin D is recommended to support bone health [72], and flexible, lightweight shoes are advocated to promote natural foot movement and reduce the risk of injury [61,63,68].

### 3.9. Pretraining Recommendations for Youth Runners

Guidelines and recommendations provided prior to initiating training are invaluable for coaches, fitness professionals, parents, and youth runners alike, serving as essential tools to ensure safe and effective training practices (Table 2).

There is no conclusive evidence, either scientific or anecdotal, indicating that distance runners need to commence training at a young age in order to achieve their peak performance. Contrary to popular belief, most elite and world-class runners did not embark to their training journey until their mid to late teenage years. Furthermore, aside from a few rare exceptions, children who set age-group records from the 5 km to the marathon distance generally did not transition into elite adult runners. The recommendation is that children refrain from starting regular and specialized training for distance running until at least the early stages of puberty, typically around ages 11 to 13 [41]. This does not mean that children under the age of 11 should refrain from participating in running events; children of all ages should be encouraged to run for fun and, most importantly, for health.

Prior to puberty, the physiological responses to training are not always correlated with performance in long-distance events. Instead, the primary determinants of distance-running performance in prepubescent children are closely tried to their physical maturity. Children who are taller, stronger, and faster tend to outperform their peers in distance races, mirroring their success in other sports such as basketball, baseball, and soccer. While numerous children may naturally exhibit high levels of aerobic fitness, allowing them to partake in low-intensity endurance activities, their capacity to produce energy for high-intensity endeavors remains restricted. A consistent finding in pediatric exercise science indicates that the anaerobic system does not reach full development until after puberty [41].

During puberty, children experience notable physical transformations, prompting a recommendation to restrict training volume and intensity. Normal pubertal development can independently enhance running performance. For example, the growth spurt of the lungs and heart enhances oxygen-rich blood delivery to muscles, thereby naturally increasing VO_2max_. Additionally, elevated growth hormone levels enable stronger muscle contractions, enhancing running speed and efficiency [41]. Youth who are physically immature and engage in high volumes of intense training face a relatively high risk of injuries, abnormal growth and maturation, and psychological burnout. Instead, it is recommended to postpone regular training, defined as more than three days a week over periods of several months, and specialized training, which entails focusing exclusively on running rather than engaging in a variety of sports and physical activities [41].

Not all developmental changes automatically lead to an improvement in running performance. Excessive training during periods of rapid limb growth can heighten the risk of muscular and skeletal injuries in children [73,74]. During puberty, bones lengthen at each end, particularly in the soft tissue called epiphyseal growth plates. These growth plates, weaker than hardened bone, become susceptible to fractures under the heavy, repetitive stresses of long-distance running [75]. Excessive training before puberty can also influence hormones in ways that may disrupt normal maturation and optimal health [76]. Under certain conditions, such as suboptimal nutrition, female runners may not produce estrogen at regular levels during puberty, resulting in delayed menarche or irregular menstrual cycles [77].

Fortunately, the majority of youth runners instinctively avoid harmful levels of training, naturally stopping before reaching their limits [41]. However, there are at least a few self-motivated youth runners who push themselves to extremes, as well as coaches and parents who push youth runners too far. In such cases, running injuries become fairly common [5]. Another concern for those who specialize in running at a young age is psychological burnout [9]. Such extensive running leaves little time for activities other than school, sleeping, and eating. When training becomes this all-consuming, it ceases to be enjoyable, leading many young people to drop out of running [41].

## 4. Conclusions

The surge in youth athlete participation in marathons and long-distance running lacks a consensus on the health and safety of younger participants. While elementary-age children can enjoy distance running safely in a well-structured environment, there is a need to establish a decision-making process for secure participation. Guiding health professionals, coaches, parents, and medical directors in conducting individualized training until the long-term consequences are thoroughly investigated is essential. Addressing various physiological and psychological aspects of health is crucial to ensure the safety of marathon running in youth. Moreover, well-organized distance-running activities at the elementary level not only have the potential to foster sustained running activity through adolescence but also to promote increased physical activity levels for a lifetime. Therefore, I strongly advocate for further research in this area.

In conclusion, Hippocrates’ timeless words resonate: “If we could give every individual the right amount of nourishment and exercise, not too little and not too much, we would have found the safest way to health”. This profound insight reminds us of the fundamental importance of balanced nutrition and exercise in achieving optimal health and wellbeing.

## 5. Suggestions for Future Research

While there is no minimum age requirement for marathon participation, individuals under the age of 18 are known to participate [9]. The unique effects of long-distance running on persons whose bodies are still growing and developing have not been fully examined and require further study.

A few articles have delved into studies focusing on the emotional health of marathon runners; they have underscored a limited understanding of the mental health aspects of youth marathon runners. Additionally, gaining insights into how marathon runners balance their everyday lives with the demands of marathon training could contribute to promoting exercise adherence in the broader population [17]. While some discussion on social and familial ramifications exists, further research is needed to provide the general population with a comprehensive understanding of how participating in a marathon may influence present and future relationships. Additionally, it is well established that intense training in children can lead to unnecessary psychological stress. Future studies could examine the long-term effects of marathon running and training at a young age on athletes’ careers during their teenage years, examining aspects such as burnout and dropout rates [17].

Another dimension of wellness noticeably absent from the literature was the impact of a runner’s career and occupational health on marathon running. This focal point should involve examinations of the relationship between marathon running and vocational responsibilities. Of particular interest is whether the skills developed during marathon running cross over into usable vocational applications [39]. Furthermore, there have been no studies examining the impact of genetics and individual predispositions on injury risk and performance in youth marathon runners.

Finally, to our knowledge, no data are available on the impact of marathons on the cardiovascular system in young adults. Future studies and research should examine this crucial aspect to provide a more comprehensive understanding of the cardiovascular effects of marathon running in the youth population [39].

## Figures and Tables

**Table 1:** Recommendation of youth runners participating in marathon races.

Point	Recommendation
1	Examine motivation for marathon running, emphasizing voluntary participation over extrinsic motivation.
2	Inform children of their right to stop participation without consequences and discuss how to communicate this choice with parents.
3	Advise parents that childhood participation in endurance running does not offer competitive advantages in later years.
4	Recommend starting with shorter events (e.g., 5 km) and gradually progressing to longer ones (e.g., 10 km, half marathon) before attempting a marathon.
5	Base readiness for running, particularly long distances like marathons, on individual growth and development rather than just chronological age.
6	Youth distance runners should rest 1 day per week, take 1–2 weeks off every 3 months, and limit participation to 9–10 months per year.
7	Recommend daily calcium (1300 mg) and vitamin D (600 IU) supplements for runners aged 9 to 18 years.
8	Encourage the use of flexible, lightweight shoes without extra cushioning and support to promote foot musculature development and encourage a forefoot or midfoot strike pattern.

**Table 2:** Pretraining recommendation for youth runners participating in marathon races.

Point	Recommendation
1	No evidence suggests that distance runners need to start training young for peak performance; most elite runners begin training in their mid to late teens. Few children with age-group records in distance running transition into elite adult runners.
2	Recommend children refrain from regular and specialized distance running training until early puberty (ages 11–13). Encourage participation in fun runs for younger children and promote running for overall health benefits.
3	Physiological adaptations to training before puberty may not correlate with performance in distance running. Physical maturity factors like height, strength, and speed are key predictors of prepubescent running performance.
4	Prepubescent children have limited energy production for high-intensity activities due to underdeveloped anaerobic systems. Anaerobic system development typically occurs post-puberty.
5	Limit volume and intensity of training during puberty as normal growth and development enhance running performance. Growth spurt in lungs and heart, and increased growth hormone levels during puberty contribute to improved running abilities.
6	Physically immature youth engaging in intense training face injury, abnormal growth, and psychological burnout risks. Suggest limiting training frequency and specializing in running only after puberty.
7	Not all developmental changes automatically enhance running performance; rapid limb growth during puberty may disrupt coordination and increase injury risks.
8	Excessive prepubescent training increases injury risks due to weaker growth plates and slower muscle development. Hormonal disruptions, like estrogen irregularities, can occur with excessive training.
9	Most youth runners naturally avoid excessive training, but some may push themselves too far, leading to common running injuries. Psychological burnout is a concern for youth runners engaging in excessive training volumes.
